# Neuropathic pain in patients with rotator cuff tears

**DOI:** 10.1186/s12891-016-1311-5

**Published:** 2016-11-02

**Authors:** Tatsuki Karasugi, Junji Ide, Toshio Kitamura, Nobukazu Okamoto, Takuya Tokunaga, Hiroshi Mizuta

**Affiliations:** 1 Department of Orthopaedic Surgery, Faculty of Life Sciences, Kumamoto University, 1-1-1, Honjo, Central Ward, Kumamoto City, 860-8556 Japan; 2 Department of Advanced Joint Reconstructive Surgery, Kumamoto University Hospital, Kumamoto University, 1-1-1, Honjo, Central Ward, Kumamoto City, 860-8556 Japan; 3 Kumamoto Orthopaedic Hospital, 1-15-7, Kuhonji, Central Ward, Kumamoto City, 862-0976 Japan

**Keywords:** Neuropathic pain, Nociceptive pain, painDETECT questionnaire, Rotator cuff tear

## Abstract

**Background:**

Recent studies have confirmed the existence of neuropathic pain (NeP) components in patients with musculoskeletal disorders. However, the presence of NeP in patients with rotator cuff tears has not been investigated thus far. Therefore, we studied the prevalence of NeP and the prognostic factors for NeP in patients with rotator cuff tears.

**Methods:**

Data were collected from 110 patients with rotator cuff tears, diagnosed by physical examination and magnetic resonance imaging, who attended an outpatient clinic between August 2013 and August 2014. The measured parameters included visual analog scale (VAS) pain scores, painDETECT questionnaire (PDQ) responses, a physical examination, and magnetic resonance imaging. To evaluate the factors associated with NeP, we performed a two-stage analysis. For univariate analysis, we used the Mann-Whitney *U* test. For multivariate analysis, forward stepwise regression was performed using factors that demonstrated statistical significance in the univariate analysis.

**Results:**

Patients were classified into three groups according to their PDQ score: an NeP group (*n* = 12; 10.9 %), possible NeP group (*n* = 33; 30.0 %), and a nociceptive pain (NoP) group (*n* = 65; 59.1 %). In the univariate analysis between the NeP group and NoP group, NeP was affected by sex (*p* = 0.034), VAS score (average pain during the past 4 weeks; *p* = 0.013), and positive Neer and Hawkins impingement signs (*p* = 0.039). In the multivariate analysis, VAS score (*p* = 0.031) was an independent prognostic factor for NeP.

**Conclusions:**

Using the PDQ, we found that 10.9 % of patients with rotator cuff tears may have NeP. The VAS score (average pain during the past 4 weeks) was a prognostic factor for NeP. Clinicians should remain vigilant for heterogeneous etiologies of pain in patients with rotator cuff tears.

## Background

Rotator cuff tear is one of the major causes of pain and dysfunction of the shoulder in the middle-aged population. According to a recent epidemiological study, the prevalence of rotator cuff tears was found to be 20.7 % in the general population, with a mean age of 58 years (range, 22–87 years), and increased with age [1]. Rotator cuff tear can lead to persistent shoulder pain and considerable disability. Although the pain caused by rotator cuff tears is generally classified as nociceptive pain (NoP), occasionally, it does not improve with anti-inflammatory medication, and continued pain becomes the main surgical indication.

The primary etiology of pain in musculoskeletal disorders is mechanical stimulation and inflammation [2]. However, recent studies have shown the clear existence of neuropathic pain (NeP) components in patients with chronic low back pain and knee osteoarthritis based on responses to the painDETECT questionnaire (PDQ) [3, 4]. Recently, Gwilym et al. used the PDQ to detect NeP in patients with impingement syndrome of the shoulder [5]. Correct identification of NeP in musculoskeletal disorders enables the introduction of the appropriate treatment for musculoskeletal pain. The PDQ is not only a simple and efficient screening tool to identify the likelihood of NeP, but also demonstrates higher sensitivity and specificity than that of other screening tools for NeP [3, 6]. Clinicians are strongly recommended to use the PDQ to assess for the presence of NeP [7]. However, the existence of NeP in patients with rotator cuff tears has not been investigated thus far. Therefore, the aim of this study was to examine the prevalence of NeP in patients with rotator cuff tears using the PDQ, and to elucidate the factors associated with NeP. We hypothesized that the etiology of shoulder pain in patients with rotator cuff tears may be multifactorial, with a mixture of nociceptive and neuropathic components.

## Methods

This study was conducted in accordance with applicable laws and regulations, including the guidelines of the Declaration of Helsinki for human experimentation. All patients provided written informed consent and the protocol and informed consent forms were approved by the local institutional review board (approval number: 1889). Two hospitals (KUH, KOH) participated in this study and used the same protocol.

### Inclusion and exclusion criteria

The inclusion criteria were as follows: patients with (1) shoulder pain, and (2) rotator cuff tear on magnetic resonance imaging (MRI) scan. The exclusion criteria were as follows: (1) moderate or severe joint degeneration according to the radiographic classification established by Samilson and Prieto [8] and musculoskeletal abnormalities including calcifying tendinitis on plain radiographs, (2) pain with cervical motion and positive results on a Spurling test or Jackson’s test during cervical spine examination [9, 10], (3) history of central or peripheral nervous system lesions, (4) diabetes mellitus, (5) prior surgery to the affected shoulder, (6) duration of symptoms less than 1 month or longer than 60 months, (7) a workers’ compensation claim, and (8) a history of medication use for NeP.

**Fig. 1:**
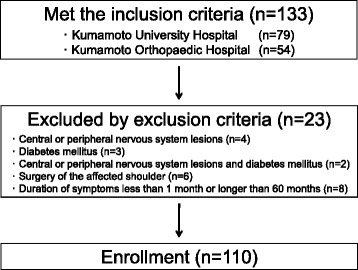
Flow chart showing the number of patients enrolled, according to our inclusion and exclusion criteria

Patients who enrolled in this study were selected from a source population of outpatients who attended one of the two participating hospitals for the treatment of shoulder pain between August 2013 and August 2014. Clinical assessment consisted of a structured interview, completion of the PDQ, a visual analog scale (VAS) for pain, a detailed physical examination, plain radiographs, and MRI scans. Among the 133 patients who met the inclusion criteria, 23 patients were excluded, leaving 110 patients enrolled in the study (Fig. 1). Demographic data and clinical features of the subjects, including age, sex, history of trauma, duration of symptoms, and VAS pain scores, are summarized in Table 1. All data were collected prospectively and analyzed retrospectively.

**Table 1:** Demographic data and clinical features of the subjects ^a^(*n* = 110)

Age (y)	65.7 ± 8.5 (46–88)
Sex (male/female)^b^	60 (54.5)/50 (45.5)
History of trauma^b^	47 (42.7)
Duration of symptoms (months)	9.9 ± 14.2 (1–60)
Visual analog scale score	
Pain at the initial visit (points)	5.3 ± 2.8 (0–10)
Most severe pain during the past 4 weeks (points)	7.4 ± 2.6 (0–10)
Average pain during the past 4 weeks (points)	5.6 ± 2.6 (0–10)

^a^Values are expressed as mean ± SD (range)

^b^Values are expressed as number of patients (%)

### PainDETECT questionnaire (PDQ)

The PDQ established by Freynhagen et al. [3, 11] was used to identify the presence of NeP. The self-administered questionnaire consists of 9 questions that address the quality of NeP symptoms; no physical examination is required. The first 7 questions address the gradation of pain, and are scored from 0 to 5 (0 = never to 5 = very strongly). Question 8 addresses the pain course pattern, scored from –1 to 1, depending on which pain course pattern diagram is selected. Question 9, for which the response is “yes” or “no”, is scored as 2 or 0, respectively, and addresses radiating pain (Table 2). The final score is between –1 and 38 and indicates the likelihood of a neuropathic component. A score of ≤ 12 indicates a low likelihood of a neuropathic component (NoP group), while a score of ≥ 19 suggests a high likelihood of a neuropathic component (NeP group). A score between these values indicates the possibility of a neuropathic component.

**Table 2:** The painDETECT questionnaire

Item	Score
Gradation of pain^a^	
Do you suffer from a burning sensation (e.g. stinging nettles) in the marked areas?	0–5
Do you have a tingling or prickling sensation in the area of your pain (like crawling ants or electrical tingling)?	0–5
Is light touching (clothing, a blanket) in this area painful?	0–5
Do you have sudden pain attacks in the area of your pain, like electric shocks?	0–5
Is cold or heat (bath water) in this area occasionally painful?	0–5
Do you suffer from a sensation of numbness in the areas that you marked?	0–5
Does slight pressure in this area, e.g. with a finger, trigger pain?	0–5
Pain course pattern	
Please select the picture that best describes the course of your pain:	
Persistent pain with slight fluctuations	0
Persistent pain with pain attacks	–1
Pain attacks without pain between them	+1
Pain attacks with pain between them	+1
Radiating pain	
Does your pain radiate to other regions of your body? Yes/No	+2/0

^a^For each question: never, 0; hardly noticed, 1; slightly, 2; moderately, 3; strongly, 4; very strongly, 5

The Japanese version of the PDQ has been validated, and has good reliability and validity according to a study by Matsubayashi et al. [7]. The study included patients with neuropathic pain related to the following pathologies: brachial plexus injury (12 patients), radiculopathy (12 patients), herpes zoster (11 patients), spinal cord injury (10 patients), diabetic or alcoholic polyneuropathies (7 patients), phantom pain (5 patients), complex regional pain syndrome (CRPS; 2 patients), carpal tunnel syndrome (1 patient), and thalamic pain (1 patient) [7]. The intraclass correlation coefficient for test–retest reliability was 0.94, and Cronbach’s alpha for the total score and main component were 0.78 and 0.80, respectively [7].

### VAS for pain

The VAS, with a range from 0 to 10 (0 = no pain to 10 = worst possible pain), was used to evaluate the current pain experienced at the initial visit, the most severe pain experienced during the past 4 weeks, and the average pain experienced during the past 4 weeks.

### Physical examination

Range-of-motion assessment included the measurement of forward elevation, lateral/scapular elevation, external rotation with the arm at the patient’s side, and internal rotation behind the back, which was recorded as the highest vertebral spinous process attained. Manual muscle tests for strength and Neer and Hawkins impingement tests were performed, as well as supraspinatus [12], infraspinatus [13], lift-off [14, 15], belly-press [14, 15], and Speed tests to evaluate the rotator cuff and biceps. All patients were evaluated at the initial visit.

### Radiographic evaluation

Anteroposterior and axillary (West Point) radiographs and MRI scans were obtained for all symptomatic shoulders.

The size of the rotator cuff tear was established based on the extent of the tear in the anteroposterior direction as measured in a sagittal oblique plane on T2-weighted MRI. Tears were then classified into 5 grades: partial (articular-sided, bursal-sided, and intratendinous tears), small (<1 cm), medium (≥ 1 cm and < 3 cm), large (≥ 3 cm and < 5 cm), and massive (≥ 5 cm). When a fluid-equivalent signal was visible or when the tendon could not be visualized in at least one section of a fluid-sensitive sequence, a full-thickness rotator cuff tear was diagnosed. MRI studies were also evaluated to assess the specific location of the rotator cuff tear. The presence of fluid in the glenohumeral joint and subacromial space was also evaluated in the coronal oblique plane on T2-weighted MRI to detect hydrarthrosis.

### Statistical analysis

To assess the association between pairs of qualitative variables, we used the Mann-Whitney *U* test for univariate analysis. For multivariate analysis, logistic regression with a forward stepwise technique was performed using factors that demonstrated statistical significance in the univariate analysis. A *P* value of < 0.05 was considered statistically significant. Power analyses were performed with EZR (Saitama Medical Center, Jichi Medical University, Saitama, Japan) [16], which is a graphical user interface for R (The R Foundation for Statistical Computing, Vienna, Austria).

## Results

### PainDETECT score

Twelve patients (10.9 %) were classified into the NeP group (score ≥ 19), 33 patients (30.0 %) into the possible NeP group (score 13 to 18), and 65 patients (59.1 %) into the NoP group (score ≤ 12) (Table 3).

**Table 3:** PainDETECT scores

Score	Number of patients (%)
−1–12	65 (59.1)
13–18	33 (30.0)
19–38	12 (10.9)

On the basis of the responses to the painDETECT questionnaire, neuropathic pain is likely with a score ≥ 19, possible with a score from 13 to 18, and unlikely if the score is ≤ 12

### Demographic data and clinical features

In the univariate analysis between the NeP group and NoP group, the female-to-male ratio was significantly higher in the NeP group than in the NoP group (*p* = 0.034), and the mean VAS score (average pain during the past 4 weeks) was significantly higher in the NeP group than in the NoP group (*p* = 0.013). Other demographic data, including age, history of trauma, and duration of symptoms, did not differ significantly between the groups (Table 4).

**Table 4:** Clinical factors associated with neuropathic pain according to univariate analysis^a^ (*n* = 77)

	PainDETECT questionnaire		*p*-value
	Neuropathic pain (*n* = 12)	Nociceptive pain (*n* = 65)	
Age (y)	70.3 ± 8.9 (58–88)	65.5 ± 8.1 (46–83)	0.117
Sex (male/female)^b^	3 (25.0)/9 (75.0)	38 (58.5)/27 (41.5)	0.034*
History of trauma^b^	5 (41.7)	30 (46.2)	0.776
Duration of symptoms (months)	11.3 ± 16.5 (1–60)	9.7 ± 13.5 (1–60)	0.439
Visual analog scale score			
Pain at the initial visit (points)	6.5 ± 2.1 (3–9)	4.6 ± 3.0 (0–10)	0.051
Most severe pain during the past 4 weeks (points)	8.2 ± 1.9 (5–10)	6.8 ± 3.0 (0–10)	0.161
Average pain during the past 4 weeks (points)	6.8 ± 1.2 (5–9)	4.8 ± 2.6 (0–10)	0.013*
Positive Neer and/or Hawkins impingement tests^b^	12 (100)	47 (71.2)	0.039*
Positive supraspinatus test^b^	11 (91.7)	51 (78.5)	0.292
Positive infraspinatus test^b^	3 (25.0)	14 (21.5)	0.792
Positive lift-off and/or belly-press tests^b^	2 (16.7)	9 (13.8)	0.562
Hydrarthrosis^b^	8 (66.7)	39 (60.0)	0.666
Size of rotator cuff tear^b^			
Partial			
articular-sided	2 (16.7)	2 (3.1)	0.053
bursal-sided	3 (25.0)	6 (9.2)	0.121
intratendinous	1 (8.3)	5 (7.7)	0.934
Small (< 1 cm)	0 (0)	9 (13.8)	0.173
Medium (≥ 1 cm, < 3 cm)	3 (25.0)	27 (41.5)	0.284
Large (≥ 3 cm, < 5 cm)	3 (25.0)	10 (15.4)	0.417
Massive (≥ 5 cm)	0 (0)	6 (9.2)	0.276
Tear location^b^			
Supraspinatus	12 (100)	64 (98.5)	0.667
Infraspinatus	4 (33.3)	21 (32.3)	0.945
Subscapularis	3 (25.0)	14 (21.5)	0.792

*Statistically significant difference between the groups (*p* < 0.05)

^a^Values are expressed as mean ± SD (range)

^b^Values are expressed as number of patients (%)

### Physical examination

In the univariate analysis, the number of positive results for the Neer and Hawkins impingement tests was significantly higher in the NeP group (100 % [12 of 12]) than in the NoP group (71.2 % [47 of 65]; *p* = 0*.*039). Other physical examination results, including the supraspinatus, infraspinatus, lift-off, and belly press tests, did not differ significantly between the groups (Table 4).

### Radiographic evaluation

In the univariate analysis, radiographic findings, including hydrarthrosis, tear location, and rotator cuff tear size did not differ significantly between the groups (Table 4).

### Multivariate analysis

In the multivariate analysis using logistic regression, we included variables that demonstrated statistical significance in the univariate analysis, specifically VAS score (average pain during the past 4 weeks), sex, and the results of the Neer and Hawkins impingement tests. The VAS score (average pain during the past 4 weeks) was found to be an independent prognostic factor for NeP (*p* = 0.031) whereas NeP was not affected by sex or the results of the Neer and Hawkins impingement tests (Table 5).

**Table 5:** Clinical factors associated with neuropathic pain according to multivariate analysis using logistic regression

Independent variable	Exp	95 % CI	*p-*value
Sex	3.871	0.853–17.569	0.079
Visual analog scale pain score			
Average pain during the past 4 weeks	0.674	0.471–0.964	0.031*
Positive Neer and/or Hawkins impingement tests		**-**	0.989

*Statistically significant difference (*p* < 0.05)

## Discussion

To our knowledge, this is the first study to utilize the PDQ in patients with shoulder pain attributed to rotator cuff tears. We found that 10.9 % of patients with rotator cuff tears may have NeP. Multivariate analysis revealed that a higher VAS score for average pain during the past 4 weeks was significantly associated with the development of NeP. Therefore, clinicians should remain vigilant for heterogeneous etiologies of pain in patients with rotator cuff tears.

There are some pharmacotherapy guidelines for NeP [17–20]. Antidepressants and calcium channel alpha 2-delta ligands are recommended as first-line therapy. Therefore, the diagnosis of NeP in patients with a rotator cuff tear is essential for optimal treatment. The clinical outcomes of pharmacotherapy for these patients needs to be elucidated.

Freynhagen et al. conducted a multicenter study and found that 37 % of 7772 patients with various forms of chronic low back pain exhibited pain that was predominantly related to an NeP component [3]. Subsequently, NeP has been reported to exist in 15 to 37.9 % of patients with low back pain on the basis of their PDQ responses [21–25]. The pathology of NeP in patients with low back pain has been attributed to nerve tissue damage generated by mechanical compression or inflammation of the nerve root due to degenerative disc disease [11]. Results from studies in animal models have also suggested that factors such as notch signaling activation [26], transient receptor potential ankyrin 1 (TRPA1) [27], and Cdh1 [28] are involved in the pathogenesis of NeP.

The PDQ has also been used to identify NeP in patients with musculoskeletal disorders such as knee osteoarthritis. NeP has been reported to exist in 5.4 to 32 % of patients with knee osteoarthritis on the basis of their responses to the PDQ [4, 29–32]. The mechanism of NeP in osteoarthritis remains unclear, but structural changes of the joint and changes in pain processing of the central nervous system have been implicated [33]. In a mono-iodoacetate-induced knee osteoarthritis study in rats, Orita et al. reported that the initial inflammatory pain state, induced by local inflammation, was followed by the gradual initiation of neuronal injury, which may have contributed to the development of NeP [34].

The pathogenesis of NeP in patients with rotator cuff tears remains unclear. There are neural mechanoreceptors such as Pacinian corpuscles, Ruffini endings, and Golgi tendon organs, as well as nociceptors such as free nerve endings in torn rotator cuff tissue and in the subacromial bursa. In the shoulder, these mechanoreceptors and nociceptors are mainly innervated by the suprascapular nerve (C5) [35]. Inflammation caused by rotator cuff tears may trigger injury of the neural mechanoreceptors and suprascapular nerve, thus leading to the gradual development of NeP. Further analysis of the interaction between rotator cuff tears and NeP is required to clarify the etiology of NeP in patients with rotator cuff tears.

This study has several limitations. First, the reliability of the PDQ for identifying NeP in patients with rotator cuff tears has not been assessed thus far. However, the original validation study included a large sample (*n* = 411) of patients with chronic pain who were recruited from 10 specialized pain centers [3]. The Japanese version of the PDQ has shown excellent test-retest reliability (intraclass correlation coefficient > 0.93) and good internal consistency (Cronbach’s alpha ≥ 0.78) [7]. Furthermore, the PDQ demonstrated excellent criterion validity when compared to an expert pain physician as the reference standard, as indicated by a high sensitivity, specificity, and positive predictive value (all > 80 %) [3]. Second, adequate statistical power to evaluate the outcomes was lacking because of the small number of patients in the study sample. According to the power analysis (type I error probability [a] = .05), the statistical power in this study was 0.703 (VAS score; average pain during the past 4 weeks), which is comparatively low. Therefore, the probability of a type I error is limited. Despite these limitations, we believe that the results of this study can be helpful to clinicians when treating patients with shoulder pain related to rotator cuff tears.

## Conclusions

By using the PDQ, we found that 10.9 % of patients with rotator cuff tears may experience NeP. Furthermore, we found that VAS score (average pain during the past 4 weeks) was a prognostic factor for NeP in these patients. This is a novel approach to an important subject and will hopefully encourage future research on the topic of neuropathic shoulder pain in patients with rotator cuff tears, as there is currently a paucity of information. We believe that the creation of an animal model with NeP and rotator cuff tears is needed to clarify the etiology of NeP in patients with rotator cuff tears. Our results indicate that clinicians should remain vigilant for heterogeneous etiologies of pain in patients with rotator cuff tears.

## Abbreviations

MRI: Magnetic resonance imaging
NeP: Neuropathic pain
NoP: Nociceptive pain
PDQ: painDETECT questionnaire
VAS: Visual analog scale
